# Outcomes of a Community Cat Program Based on Sterilization of Owned, Semi-Owned and Unowned Cats in a Small Rural Town

**DOI:** 10.3390/ani14213058

**Published:** 2024-10-23

**Authors:** Jacquie Rand, Abithaswathi M. Saraswathy, Joy Verrinder, Mandy B. A. Paterson

**Affiliations:** 1 Australian Pet Welfare Foundation, Brisbane, QLD 4064, Australia; a.munirajsaraswathy@uqconnect.edu.au; 2 The University of Queensland, Faculty of Science, School of Veterinary Science, Gatton Campus, Gatton, QLD 4343, Australia; mpaterson@rspcaqld.org.au; 3 Animal Welfare League, Coombabah, QLD 4216, Australia; 4 Royal Society for Prevention of Cruelty to Animals, Wacol, QLD 4076, Australia

**Keywords:** urban cat management, sterilization, community cat program, euthanasia, one welfare, enforcement-based cat management, TNR, return to field, human wellbeing, mental health

## Abstract

Free-roaming cats in urban and peri-urban areas and in rural towns pose significant challenges due to their nuisance behaviors, threats to wildlife, and potential disease transmission. Traditional management methods often involve trapping and euthanizing many of these cats, which is not only costly but also distressing for the shelter staff involved, and have failed to reduce numbers of impounded cats or cat-related complaints. Our study aimed to evaluate the effectiveness of a Community Cat Program that provided free sterilization, microchipping, and preventative veterinary care for all owned, semi-owned, and unowned cats in a small rural town with high cat impoundments in Ipswich, Queensland. The program was instigated by the Australian Pet Welfare Foundation in collaboration with the Royal Society for the Prevention of Cruelty to Animals, Queensland and the Animal Welfare League, Queensland, and it was funded by various organizations including Fondation Brigitte Bardot. Data from 2017 to 2023 were analyzed to assess the impact of the program on cat intake and outcomes in local shelters. A total of 308 cats were sterilized, representing 94 cats per 1000 residents over 3.4 years, and in the third year, this was associated with a 60% decrease in cat intake, an 85% reduction in numbers euthanized and 39% fewer cat-related calls to the local council. These findings suggest that high-intensity, targeted sterilization programs significantly decrease shelter intake and euthanasia, and thus alleviate the psychological burden on staff. These programs are a humane and effective alternative to traditional cat management, and benefit animal welfare as well as community wellbeing. This is aligned with a One Welfare approach, which enhances the well-being of animals, humans, and their environments. Legislative changes are required to support these programs to effectively decrease the numbers of free-roaming cats in cities and towns and the issues they cause, while protecting human wellbeing.

## Simple Summary

In rural towns of Australia, free-roaming cats are often in high numbers on a human population basis and frequently cause nuisance issues. Traditional methods of managing these cats often consist of trapping and euthanizing those not adopted, which can be costly and distressing for the people involved and not aligned with community preferences. Our study focused on a pilot Community Cat Program in small rural town of Rosewood, Australia. The program provided free sterilization, microchipping, and preventative health care for all cats in the area. The program aimed to reduce the number of cats entering shelters and being euthanized. In the third year of the program, cat intake had decreased by 60%, numbers euthanized by 85%, and there were 39% fewer cat-related calls to council. These results highlight the rapid effectiveness of targeted sterilization programs in reducing euthanasia and cat-related complaints. These programs are a humane and sustainable method of managing free-roaming cat populations and are aligned with a One Welfare approach which aims to optimize the wellbeing of humans, animals, and their environments.

## 1. Introduction

In many countries, free-roaming cats in urban and peri-urban areas and in rural towns create significant challenges [1,2,3,4]. Frequent issues include nuisance behaviors such as fighting, yowling, defecation and urination, concerns about threats to wildlife, and potential disease transmission, including zoonotic diseases [1,2,3,4]. Often, these concerns take priority over the cats’ welfare, leading to a focus on enforcement-centered cat management, often resulting in lethal outcomes [1,5,6].

Rural towns in Australia typically have high per capita intake of cats into the municipal animal facility (pound) or their service provider [7], which is typically the closest veterinary practice if there is no animal welfare shelter in the vicinity. This high cat intake is likely the result of lower socioeconomic levels and less accessibility to affordable veterinary care [8]. There are limited options for adoption because of the small population, and therefore the local veterinarian and their staff are frequently tasked with euthanasia of healthy and treatable cats and kittens, which may cause discord amongst some staff because of the negative impact on job satisfaction and mental health [9,10]. There is a growing advocacy for a One Welfare approach to domestic cat management, which seeks to balance and enhance the wellbeing of animals, humans, and their physical and social environments [1,4,11,12,13]. By improving animal welfare, the welfare of humans and the environment also benefits.

In Australia, management of free-roaming cats is based on a legislative approach both at the state and local government levels. Enforcement is typically carried out by animal management officers (AMOs, called animal control officers in the USA) who are employed by the local government (council). They trap and impound offending cats or provide complainants with a cat-trap, and the trapped cat is then delivered to the pound or a service provider, usually an animal welfare agency or the local veterinary practice [6].

State and/or local government laws aimed at managing urban cats typically require owners to microchip, register (license) with the local or state government, and confine cats to their properties, and in some locations to sterilize them [14,15,16]. Owners with more than two or three cats are typically required to obtain and pay for an excess cat permit [15,16]. Most free-roaming and impounded cats emanate from lower socioeconomic neighborhoods [6], where compliance with laws regarding pet cats is low due to the costs involved. Many cats are acquired as strays or free from friends and family, and the cost of sterilization, microchipping, and registration is prohibitive [17]. In addition, rental properties including government housing typically do not have suitable cat-proof fencing and the cost of installing enclosures or fencing is prohibitive and may not be allowed under the tenancy agreement [18].

Most of the cats trapped in response to complaints are not identified with a microchip or collar and tag [19]. The majority are stray cats fed by compassionate people, and they can be considered semi-owned cats or are unidentified owned cats [20,21,22,23]. Compliance and enforcement-based approaches are ineffective because there is no identifiable person to issue an infringement notice or fine to [6].

Only 5% to 7% of cats entering facilities managed by local governments (pounds) and animal welfare organizations are reclaimed by their owner, with the reminder either rehomed or euthanized [24,25]. This trap–adopt/kill management leads to the unnecessary euthanasia of healthy and treatable cats and kittens. In Australia, 33% of the cats that entered shelters and municipal animal facilities in 2018 to 2019 were euthanized [24]. This situation is psychologically distressing for animal management officers [6] and shelter and pound staff who are responsible for euthanizing numerous healthy and treatable cats and kittens [9,26,27,28,29], as well as the people caring for these cats [30]. Cat management also imposes financial burdens on local governments and animal welfare groups [6].

In Australia, cats are often categorized as feral or domestic, with feral cats defined as cats having no reliance on humans and living and reproducing in the wild, separate from humans [31,32,33]. The Royal Society for Prevention of Cruelty to Animals (RSPCA) recommends that cats be classed as domestic if they live in the vicinity of humans and depend wholly or partially on humans for survival [31]. By this definition, free-roaming cats in urban and peri-urban areas, in rural towns, and around farm buildings are domestic cats, and they are either owned, semi-owned (fed by one or more people who do not perceive ownership), or unowned (obtain food from humans unintentionally) [31]. If unidentified with a microchip or collar and tag, they are considered stray cats [31,32,33]. However, under various state governments’ legislation, the distinction between feral and domestic is poorly defined. For example, the Queensland Biosecurity Act 2014 has specific provisions regarding the classification of cats [34]. According to the Act, only cats that are owned are recognized as domestic cats [34], with owners being defined as those who usually keep the cat, perceive them as personal property, or are the registered owner under a local council if required [14]. All other cats, even those in urban or peri-urban areas, are categorized as feral or “restricted matter”, including stray or community cats, and the *Act* imposes various restrictions, or mandates actions concerning these cats. For instance, cats identified as “restricted matter” cannot be fed, moved, adopted, or sold [34,35]. However, cats in a pound or shelter that are friendly, even without identification, are considered to have been owned (therefore domestic) and are allowed to be adopted. In contrast, trapped cats are often stressed and fearful, and may appear timid, shy, aggressive or unfriendly and, under the Queensland Biosecurity Act 2014, based on lack of a microchip or collar and behavior in a trap or soon after admission, are frequently misclassified as feral and euthanized, without time to acclimate to the shelter environment, which may take eight or more days [36,37].

Trap–neuter and return (TNR) is illegal in all states of Australia related to various legislations relevant to abandonment, biosecurity, and requirements to contain cats to the owners’ property [22,34,38,39]. Also illegal is return to field (RTF), whereby healthy, poorly socialized cats which are unlikely to be adopted are sterilized and returned to where they were found on the premise that there is someone feeding them.

A recent study [6] reported marked decreases in complaints, impounded and euthanized cats and kittens, as well as costs to the local government associated with a free cat sterilization program. The program targeted low-socioeconomic areas with high volumes of cat-related calls and intake into the pound and contracted shelter. The program focused on providing free sterilization, microchipping and registration (licensing) for owned cats and helping semi-owners take ownership of the cat(s) they were caring for. It was initiated and managed by the AMOs in response to negative impacts of traditional cat management on their job satisfaction and mental health.

**Figure A1:**
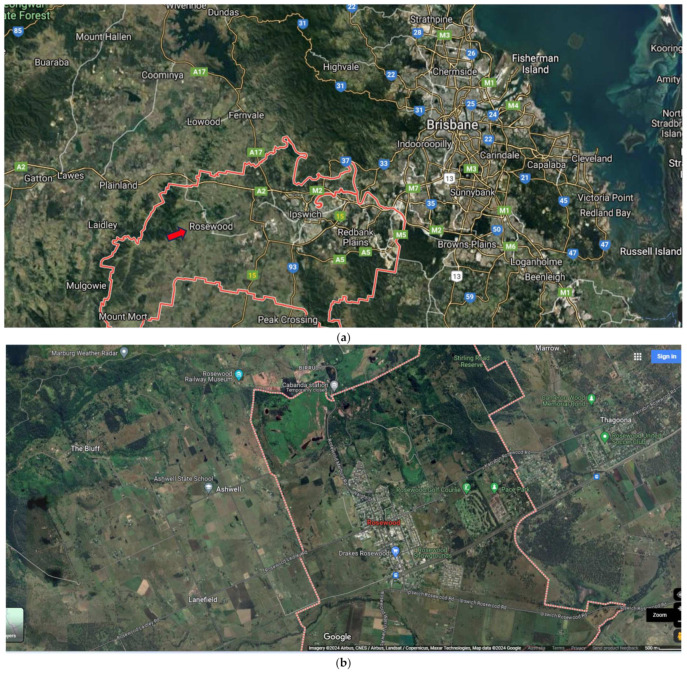
(**a**) Map of city of Ipswich showing location of Rosewood (arrow) to the west of the central business district of Ipswich, and to the east the city of Brisbane (2.5 million residents in 2021 census). (**b**) Map of rural town of Rosewood in the city of Ipswich showing rural land surrounding urban area.

In 2020, the Australian Pet Welfare Foundation (APWF) initiated a Community Cat Program based on offering free sterilization, microchipping and preventative health care for all cats in a small rural town (Rosewood) with approximately 3000 residents within the local government (council) area of the city of Ipswich, Queensland (Figure A1a,b). The program aimed to reduce shelter intake and euthanasia of cats, and the subsequent negative impact on staff involved in euthanizing healthy cats and kittens [9,10]. It also aimed to decrease the number of free roaming cats and the negative impacts they cause including complaints related to nuisance behaviors. For the city of Ipswich in 2016–2017, the average intake of cats into the receiving shelter and pound operated by RSPCA Qld was approximately 15 cats/1000 residents compared to the Queensland state average of 7 cats/1000 residents [24]. Rosewood intake exceeded 20 cats/1000 residents (calculated based on the human population of 2834 in 2016) [40]. Rosewood is surrounded by land where the primary industries are dairy farming and coal mining, but these have declined in recent years [41]. It is classified as rural residential because it is on rural land and located outside the urban development boundary for the city of Ipswich [42]. Based on the 2021 census [43], the locality of Rosewood had 53.4% females and 46.6% males, with a median age of 39 years, 1 year above the national median of 38. Most (83.5%) people living in Rosewood were born in Australia. The Socio-Economic Indexes for Areas (SEIFA) reflects people’s access to material and social resources and their ability to participate in society [44]. For Rosewood, the SEIFA index is 969, which is lower than the averages for the Ipswich local government area (1052) and below the national average for Australia (1000) [43]. Reflecting this lower SEIFA index, the median individual weekly income in the last census (2021) was below the Australian average (A$636 versus A$805), a higher proportion of the population lived in rental properties (41% versus 30.6%), and a higher proportion of renter households had rent repayments greater than 30% of household income (38.4% versus 32.2%). In the city of Ipswich, Queensland, state government legislation requires microchipping of cats between 8 and 12 weeks of age, and a local bylaw (ordinance) requires cats to be contained to the owner’s property and a permit obtained to keep more than two cats [15].

The aim of this study was to report preliminary outcomes over 3 years of a pilot Community Cat Program based on free sterilization, microchipping, and preventative health care for all owned, semi-owned, and unowned cats in a small rural town. Other ongoing research associated with the program involves evaluating numbers of free-roaming cats over 5 years using camera traps.

## 2. Materials and Methods

**Figure A2:**
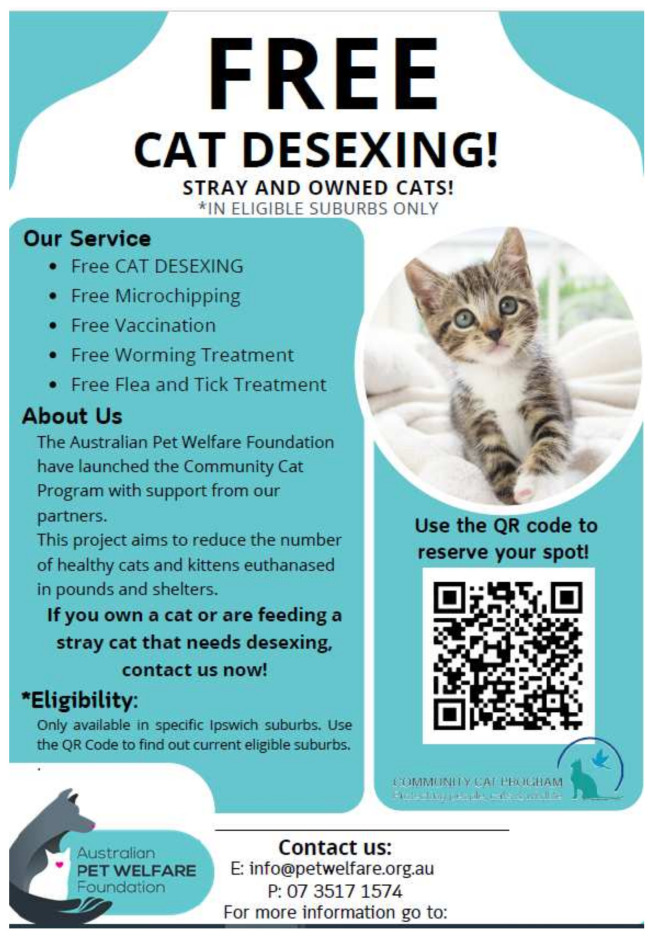
Flyer advertising program and used in letter box drops.

A Community Cat Program was implemented in Rosewood (census population 2834 in 2016 and 3263 in 2021) [40,43]. In this program, free sterilization, microchipping, vaccination, endo- and ectoparasite control, and veterinary care were offered for all cats living in Rosewood (Figure A2). The program was an initiative of the Australian Pet Welfare Foundation (APWF) in collaboration with the RSPCA Queensland (RSPCA Qld) and the Animal Welfare League, Queensland (AWLQ) with funding and in-kind support from multiple organizations, including the Fondation Brigitte Bardot (Paris, France) for sterilizations, MSD Animal Health (Macquarie Park, NSW, Australia) for vaccinations and parasite control, Central Animal Records (Keysborough, Australia) for microchip registration of cats, and Neighborhood Cats (New York, NY, USA) for cat traps and expert advice.

Cats that were semi-owned, and where the carer was unable or unwilling to take ownership of the cat(s) but wanted to continue to provide care for them were considered “restricted matter”, and were sterilized, microchipped, and ear-tipped (approved by the Queensland Government under a Department of Agriculture and Fisheries Scientific Research Permit No. PRID000825). An Animal Ethics Approval (Permit Number 2019/AE000207) from the University of Queensland’s Research Ethics and Integrity Unit covered the cats in this study. For these restricted matter cats, no owner name or contact details were registered on the microchip database, but in place of the owner’s name was Rosewood Community Cat, and the APWF name and phone numbers were listed as the secondary contact details, including the personal mobile number of one author (J.R.). Return to field (RTF) (shelter–neuter and return) cats were managed similarly. Microchip number, contact details, and address (or street name for RTF cats) for the carer were recorded on an internal database accessible 24/7 by relevant APWF, RSPCA Qld, and AWLQ staff.

Outreach staff (community liaison officers) were provided by RSPCA Qld and AWLQ to organize sterilization appointments, client consent processes, and transport of cats to and from the veterinary clinics for owners/carers in need of assistance. These staff also facilitated finding owners or carers of unsterilized cats through door-knocking, social media, letter box flyers (Figure A2), and community organizations. They also assisted with trapping cats for semi-owners and returning impounded cats to their home location after sterilization and microchipping (RTF). Where cats were at risk of impoundment associated with nuisance complaints, they assisted complainants in keeping cats off their property by providing a free loan of water deterrents. At one multi-cat site (colony), community volunteers built cat-proof fencing around the property to obviate complaints from neighbors regarding wandering cats.

Initially, all sterilizations (57 cats) were performed by Rosewood Veterinary Services for A$175/female cat (plus a A$50 to A$100 pregnancy surcharge) and A$125/male cat, which was funded by a grant from PetStock Foundation [45]. When these funds were expended, RSPCA Qld provided sterilization services at their domestic-animal clinic at Wacol, funded by a grant from Fondation Brigitte Bardot for A$46.89 per cat. APWF paid for additional medical or surgical treatment at the time of surgery if it was treatable and considered a welfare issue for the cat.

The local government animal facility (pound) at Ipswich was managed by RSPCA Qld until October 2021, after which the contract changed to AWLQ. However, approximately 25% of cats emanating from the city of Ipswich in 2022 and 2023 were directly admitted to the RSPCA Qld shelter at Wacol (approximately 25 km from the Ipswich pound), mostly from the general public. In addition to a change in service provider, other challenges during the study period included reduced services of the council pound (animal facility) in 2020 due to the COVID-19 pandemic and associated closure of the RSPCA Qld shelter at Wacol for 5 months (18 March to 27 June 2020, and 27 August to 19 September 2020). In addition, flooding of the pound in February 2018 and again in 2022 [46], resulted in closure of the pound to the public and evacuation of animals to an evacuation center, necessitating a reduction in services.

### 2.1. Data

The city of Ipswich provided data for cat-related calls to council for each calendar year from 2017 to 2023, and the reason for the call (loan of trap cage, roaming cat, excess cat numbers, cat enclosure/fencing, cat noise nuisance). For data related to shelter and pound admissions and outcomes for cats, RSPCA Qld and AWLQ records from 2017–2023 were exported from ShelterMate© version 4.53.1 [47], the data management system used by both organizations [48] and imported into Microsoft Excel for data cleaning and analysis. Data for each admission included cat identification number, admission date, age, and admission source. Admission sources were categorized as “stray” (subcategorized as from a member of the public or from the council animal management officers (“council”)), “owner surrender”, “ambulance” (RSPCA Qld’s animal ambulance service), “humane officer” (cats seized by RSPCA Qld inspectors under Queensland animal welfare legislation), “euthanasia request” (cats surrendered for euthanasia, but allowing rehoming if possible), or “shelter offspring” (born in care). For analysis, sources with small numbers of cats were amalgamated and classified as “other” and included “dead on arrival”, “emergency boarding”, “bequest in shelter” (left by someone’s estate), and “evacuation” (cared for during an emergency). Cats that were classified as “return” (cats returned within 30 days of adoption) were only included as an admission on first entry to the facility, and were not included as an admission when returned. Also excluded from admissions for target and non-target areas were cats collected by animal management officers (AMOs) from veterinary clinics where the suburb of origin was not recorded. This was particularly problematic for Rosewood because there were many surrounding rural suburbs not serviced with veterinary clinics, and cats from these surrounding suburbs were taken by the public to the Rosewood Veterinary Clinic and collected by AMOs and impounded, often without the suburb of origin being recorded.

Each admission was classified by outcome as reclaim (returned to owner), rehome (adopted), transfer (to a rescue group), euthanasia (humanely killed), died in care, or “other” (e.g., escaped or stolen). Admissions where the cat remained under RSPCA Qld or AWLQ care, including foster care, awaiting surgery, and awaiting adoption at the end of the reporting period, were classified as “in-care”. Cats moved between sites had their outcome recorded for the admission site.

### 2.2. Statistical Analyses

The unit of analysis was individual admission, where each cat’s entry into an RSPCA Qld or AWLQ shelter constituted one admission, ending with the cat outcome as described above. Cats could have multiple admissions and all admissions, except “returns”, and cats with no suburb of origin recorded were included for descriptive analyses. Human population size for the relevant local government area was used from the census data from 2016 and 2021 and used to calculate admissions and outcomes per 1000 residents [49,50].

Data from 2020 are shown but were not used in analysis because the contracted shelters were closed for nearly 5 months because of COVID-19 restrictions, reducing intake. Impact of the program was assessed by calendar years 2021 to 2023 for the number of cats admitted, reclaimed, rehomed, and euthanized, and for the percentage of impounded cats that were reclaimed, rehomed, and euthanized. Numbers of cat-related calls to council from 2017–2023 were also analyzed for the target town (population 3263) and the non-target suburbs (population 130,626) for the city of Ipswich. Suburbs where the Community Program was started later than Rosewood were not considered target or non-target suburbs (population 100,120) and were excluded from analysis.

## 3. Results

### 3.1. Numbers of Cats Sterilized and Their Characteristics

Following implementation of the program in August 2020 through to the end of December 2023, 67, 98, 85, and 58 cats were sterilized per calendar year representing 21, 30, 26, 18 cats/1000 residents per year based on 2021 census data (3263 residents) [43]. A total of 308 cats were sterilized representing a cumulative total of 94 cats/1000 residents over the 3.4 years of the program (average 27.8 cats/1000 residents per year). Of cats sterilized during the program (308), 74% (227) were owned cats, 10% (31) were semi-owned cats that became owned at the time of sterilization and microchipping, 11% (34) remained semi-owned at multi-cat sites, 0.6% (2) were RTF (returned to field) and 4.5% (14) were kittens mainly from one multi-cat site that were rehomed through the RSPCA Qld. Of the cats microchipped to an owner at the time of surgery (258), 88% (227) were owned and 12% (31) were not considered the carers’ property at the time they first started feeding them. 

**Figure A3:**
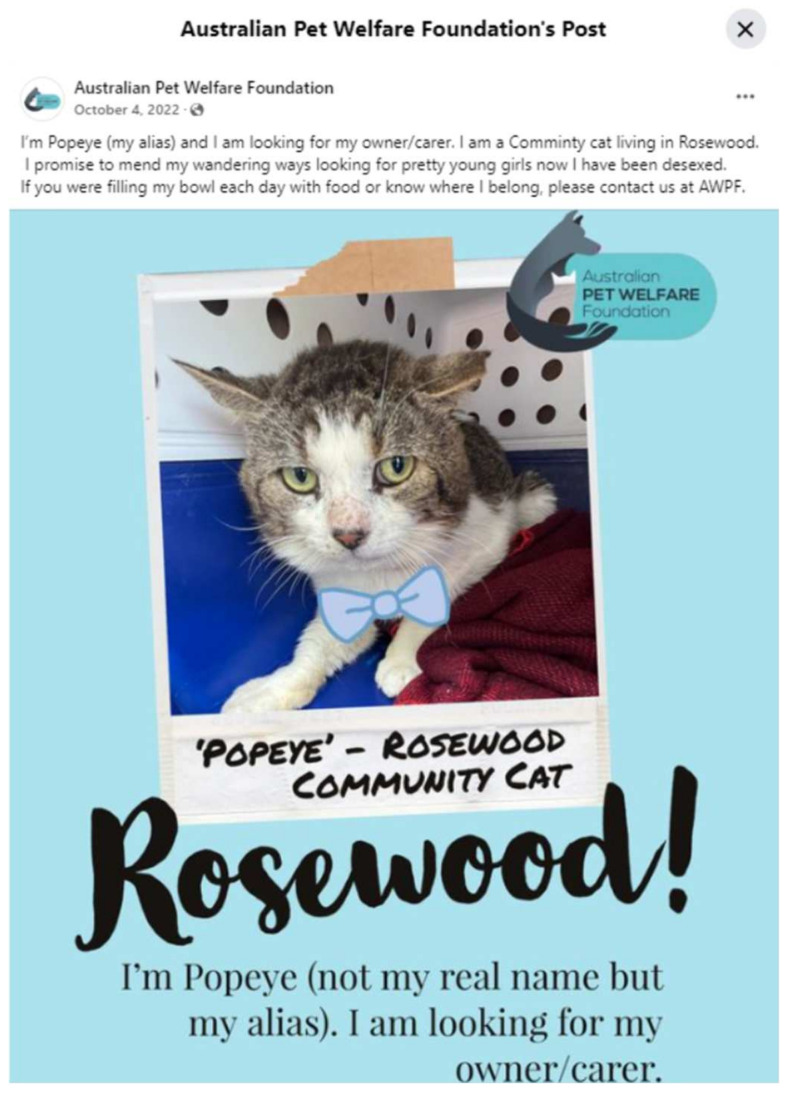
Facebook post relating to “Popeye” who was returned to field (RTF).

Of the cats which remained “restricted matter” (36 cats), 34 remained semi-owned being cared for by known carer(s). One additional female cat that was part of the one of the multi-cat sites, was subsequently found to be an unidentified owned cat, and lived behind the multi-cat site on an adjacent parallel road. After being contacted by the owner, the microchip contact details were changed to the owner (cat not included in number of restricted matter cats). All of the semi-owned cats (*n* = 34) were at three multi-cat sites consisting of 7, 12 and 15 cats. The largest site was a dairy farm. A minority of the restricted matter cats (*n* = 2) were cats that were impounded and were healthy but considered unsuitable for rehoming because of behavior assessed over 4 days to 2 weeks. They were sterilized and microchipped before they were returned to their home location (RTF, *n* = 2) on the premise that someone was feeding them. Both were found by members of the public the following day and taken to Rosewood Veterinary Services located less than a kilometer from where they had been returned. They were again impounded at the shelter, and therefore included twice in intake data in 2022. One cat was then returned to one street closer to where he was suspected to have originated. Letterbox flyers and Facebook posts (Figure A3) were used to inform the community of his presence and he was not impounded again. The second RTF cat was subsequently adopted from the shelter.

### 3.2. Cat Admissions—Number and Sources

**Figure 1:**
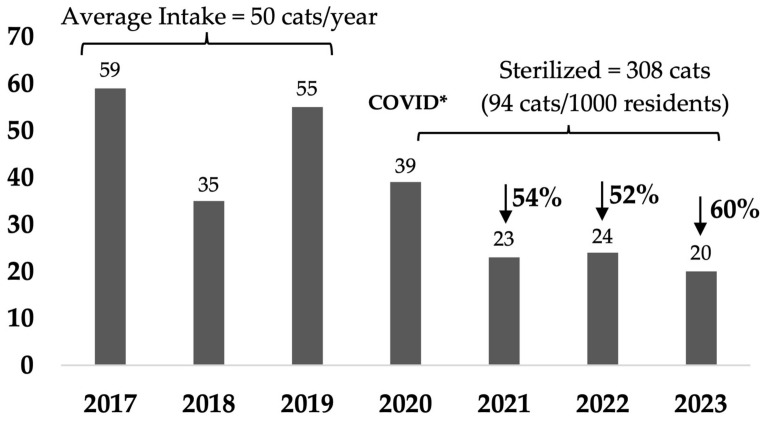
Number of total admissions from the target suburb Rosewood into RSPCA Queensland and AWL Queensland shelters between the years 2017 and 2023. * The shelter was closed for 5 months due to the COVID-19 pandemic in 2020. The Community Cat Program started August 2020.

**Figure 2:**
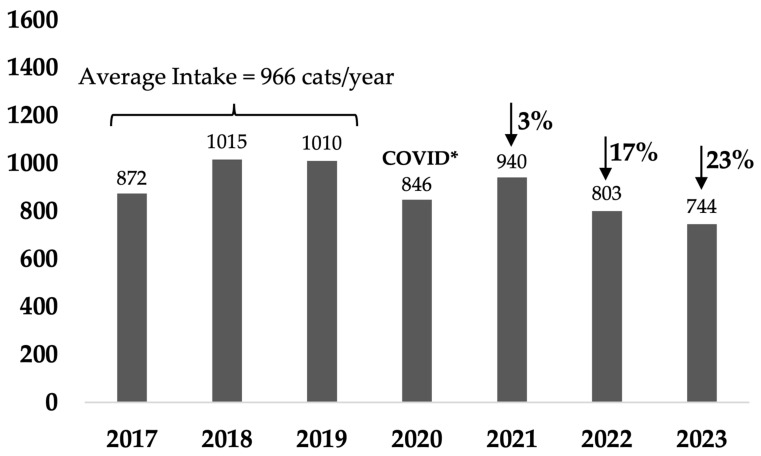
Number of total admissions from the non-target suburbs into RSPCA Queensland and AWL Queensland shelters between the years 2017 and 2023. * The shelter was closed for 5 months due to the COVID-19 pandemic in 2020.

Compared with the average intake of the three full calendar years before the program started, i.e., 2017–2019, cat admissions from Rosewood decreased by 54% by the end of the first full calendar year (January–December 2021) after 51 cats/1000 residents were sterilized over 17 months from August 2020, with subsequent decreases of 52% and 60% in Years 2 and 3 (Figure 1 and Table A1a). This represented a decrease in intake from an average of 17.6 cats/1000 residents (population 2834) over the baseline 3 years to 6 cats/1000 residents in Year 3 (population 3263). In comparison, the non-target suburbs intake decreased by 3% in the first year and then by 17% and 23% in subsequent years, with a decrease in intake from an average of 8.5 cats/1000 residents over the baseline 3 years to 5.7 cats/1000 residents in Year 3 (Figure 2 and Table A1).

**Figure 3:**
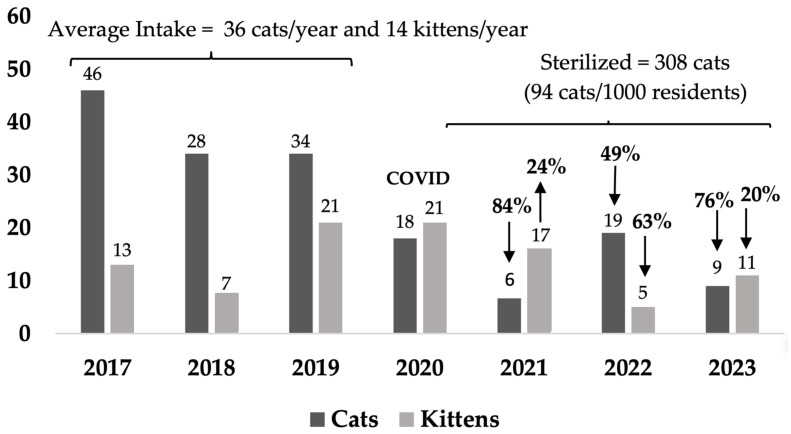
Number of cat and kitten admissions from the target suburb Rosewood into RSPCA Queensland and AWL Queensland shelters between the years 2017 and 2023. The Community Cat Program started August 2020.

**Figure 4:**
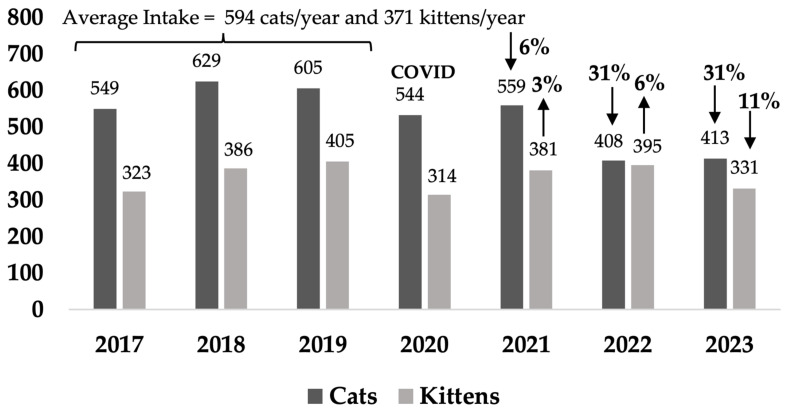
Number of cat and kitten admissions from the non-target suburbs into RSPCA Queensland and AWL Queensland shelters between the years 2017 and 2023.

When changes in sources of cats were examined over time, during the COVID pandemic in 2020–2021 and continuing after the change in service provider, intake of owner-surrendered cats was minimized in the target and non-target areas, primarily of adult cats to reduce shelter overcrowding (Table A2). In addition, there was a change in source of stray cats with a marked decrease in stray cats being received from AMOs and an increase in stray cats from members of the public (Table A1b,d). Notably, in the target area, kitten intake (all sources) decreased by 43% averaged over the last 2 years from the baseline average, but not the non-target area (Figure 3 and Figure 4, Table A3), where there was an average decrease of 2% over the last 2 years.

### 3.3. Outcomes for Cats

**Figure 5:**
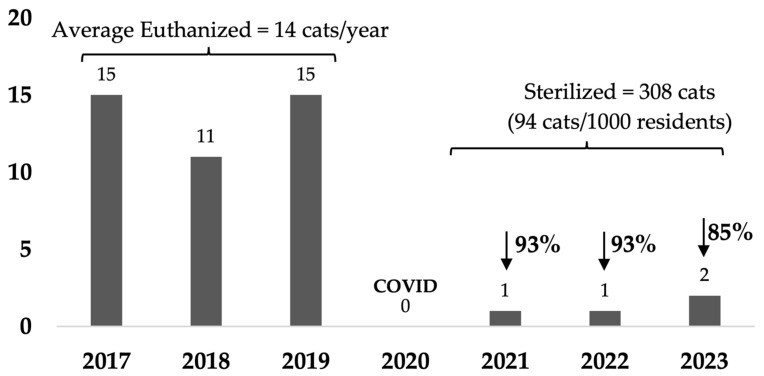
Number of euthanized cats from the target suburb Rosewood in RSPCA Queensland and AWL Queensland shelters between the years 2017 and 2023. The Community Cat Program started August 2020.

**Figure 6:**
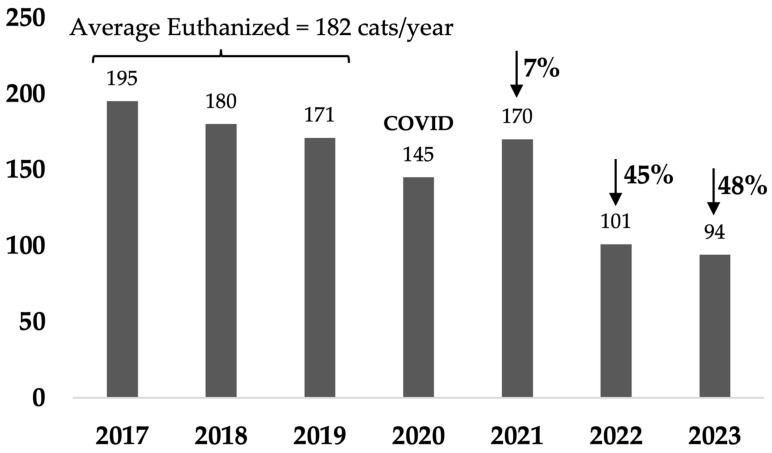
Number of euthanized cats from the non-target suburbs in RSPCA Queensland and AWL Queensland shelters between the years 2017 and 2023.

Cats reclaimed by owners represented a small proportion, with an average of only 7% reclaimed over the last 2 years of the program in the target area (10% in final year) and an average of 9% in the non-target area, which did not change over the 7 years that data were available (Table A4). The proportion of intake that was adopted was 81% in the target area and 73% in the non-target area, averaged over the last 2 years of the program. This was an increase in average percentage of intake adopted from a baseline of 56% in the target area and 63% in the non-target areas. However, total numbers adopted decreased by 38% in the target and 5% in the non-target areas averaged over the last 2 years, reflecting the greater decrease in intake of cats in the target area and hence fewer cats available for adoption. Numbers of cats euthanized in the target area (Figure 5) decreased by 89% over the last 2 years of the program compared with baseline, and euthanized cats constituted an average of 7% of intake. In contrast, in the non-target areas, the numbers of euthanized cats decreased by 46% averaged over the last 2 years, and euthanized cats constituted an average of 13% of intake (Figure 6). In the third year, cats euthanized per capita in Rosewood had decreased from a baseline of 4.9 cats to 0.6 cats/1000 residents, and in the control suburbs the number decreased from 1.6 to 0.7 cats/1000 residents compared to the Queensland average of 1.6 cats/1000 residents in 2018–2019 [24].

### 3.4. Cat-Related Calls to Council

**Figure 7:**
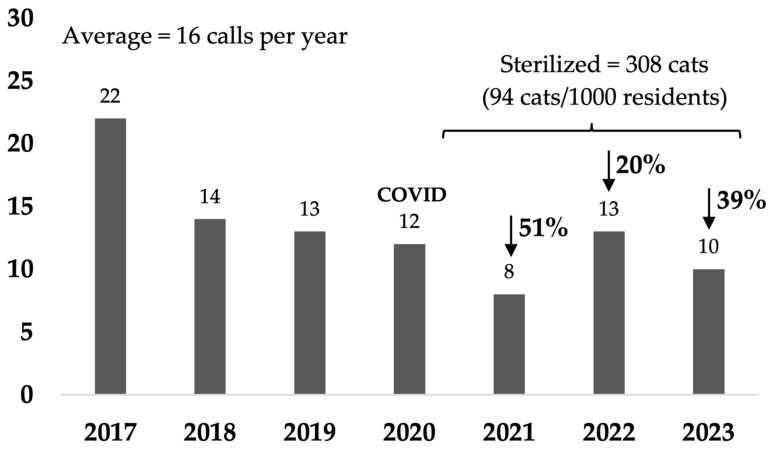
Number of cat-related calls to council (local government) emanating from the target suburb Rosewood between the years 2017 and 2023. The Community Cat Program started August 2020.

**Figure 8:**
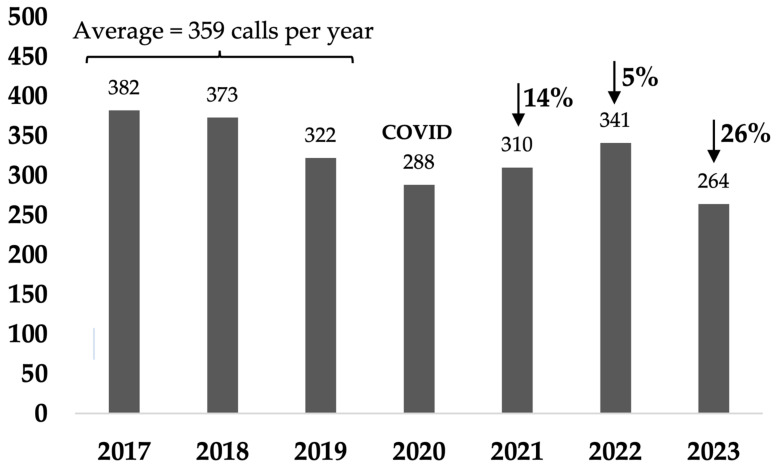
Number of cat-related calls to council (local government) emanating from the non-target suburbs between the years 2017 and 2023.

In Rosewood, cat-related calls decreased by 39% in the last year compared to the baseline (10 calls versus average baseline of 16.3 calls per year) (Figure 7 and Table A5a). In the non-target areas, cat-related calls decreased by 26% in the last year compared to the baseline average of 359 calls per year (Figure 8 and Table A5b). The major reason for cat-related calls in both target and non-target areas were calls requesting the loan of cat traps, accounting for 70% and 79% of all cat-related calls in the last year in the target and the non-target areas, respectively (Table A5a,b).

### 3.5. Program Costs

The total cost for the 308 cats sterilized was A$22,188 (average A$72.04/cat), which represented an average annual cost over 3.4 years of A$6536 or A$2/resident per year. The initial cost for sterilizing 57 cats at Rosewood Services was A$9200 which averaged A$161/cat and included occasional antibiotics administered and a pregnancy surcharge for some female cats of A$50 to A$100. This was in addition to the standard cost of A$175/female cat and A$125/male cat. Assistance for sterilization was also provided by Greencross Vets (3 cats probono) and AWLQ (5 cats probono, which included 2 restricted matter cats). For the 243 cats sterilized at RSPCA Qld, the cost for a male or female cat was A$46.89, which was the amount the Fondation Brigitte Bardot paid per cat (30 euro/cat). The cost for the 243 cats sterilized at RSPCA Qld was A$12,987 which was an average cost of A$53.45 and included some incidental costs such as antibiotics or dental extractions (average A$6.56/cat).

Other additional contributions by the RSPCA Qld included a community liaison officer who varied from part- to full-time and some administrative assistance for booking cats and recording data. After October 2021, AWL Qld also provided some assistance from two part-time community liaison officers. In addition, a vehicle for transporting cats and trap cages was provided by APWF to AWL Qld and was funded by the Gambling Community Benefit Fund. Box and drop trap cages were provided by grants from Neighborhood Cats (USA). Ipswich City Council also purchased new box traps for use by both council and AWLQ, and AWLQ purchased additional drop traps for catching cats for sterilizing who were unable to be caught using box traps. Vaccination and endo- and ecto-parasite control (Bravecto Plus, MSD Animal Health, Murarrie QLD, Australia) was provided pro bono by MSD Animal Health. Central Animal Records waived the cost of registration of the owner or carers contact details on their database, provided the microchips were purchased from them at a price of A$5/chip, paid for by APWF donors.

## 4. Discussion

Management of cats in Australia occurs typically via a traditional impoundment model where problematic free-roaming cats are trapped, and those not reclaimed by owners are either rehomed or euthanized [24]. This has widespread negative impacts on AMOs involved in trapping and transporting cats where euthanasia is the likely outcome [6], as well as shelter staff and community members caring for cats who are trapped and killed [9,26,27,28,30,51]. More recently, management based on sterilization has been advocated [52], particularly internationally, and is traditionally based on TNR which is illegal in Australia [22]. Our study reports preliminary results of a pilot program based on sterilizing owned and semi-owned cats, and cats for whom no owner or carer was found, in a small rural town in the city of Ipswich, Queensland. Key findings were that intake decreased by 60%, euthanasia by 85%, and cat-related calls by 39% in the third year after sterilizing a total of 94 cats/1000 residents over 3.4 years, most of which were owned cats, for an annual average cost of A$6526 for sterilization surgeries.

### 4.1. Numbers of Cats Sterilized

The number of cats we aimed to sterilize in our pilot program was based on prior research from USA. For example, in Florida, when 60 cats/1000 residents were sterilized each year for two years, intake was decreased by 66% in the second year in the target area of 20,000 residents [53]. Of those sterilized, 52% were returned to the original location or relocated to other sites after sterilization (TNR) [53]. However, 47% of the cats were rehomed or transferred to a rescue group and were not included in the shelter intake in the target area [53]. Another study reported a 42% decrease after 30 cats/1000 residents were sterilized in a combined TNR and RTF program [54]. However, in another study of six cities in the USA ranging in population from 200,000 to 1.8 million, an average of 5.4 cats/1000 residents were sterilized each year over three years, and shelter intake decreased by a median of 32% [55]. In their program, trapping and sterilization was microtargeted to locations of cat-related complaint calls, kitten intake, or based on AMO knowledge. The greatest decrease in intake occurred in Columbus, which implemented the “red flag” model where animal control officers utilized prior knowledge and shelter data to target “hot-spot” areas, reducing intake by 45% [55]. Thus, we elected to aim for a sterilization rate 30 cats/1000 residents per year for 3 years, based on limited information on the minimum number to be sterilized per year to achieve a measurable impact over 1 to 3 years, that the human resources and budget were limited, and the city of Ipswich council (elected officials) initially ruled no in-kind or financial support was to be provided by the city. In the first two whole calendar years of the program, we sterilized 30 and 26 cats/1000 residents, and in the final year, as demand decreased, only 18 cats/1000 residents. Sterilization, microchipping, vaccination, and parasite control were provided free with support of our many partners.

#### Factors Influencing Impact of Numbers Per Capita Sterilized—AMO Support

Recent Australian data from the city of Banyule, Victoria reported that cats impounded by AMOs decreased by 68% over 4 years in three target suburbs following a cat sterilization program where an average of 4.3 cats/1000 residents were sterilized [6]. Community engagement was led by an AMO committed to an assistive rather than an enforcement-based approach to urban cat management [6]. Sterilization was mainly microtargeted to locations of cat-related complaints within three suburbs that had the highest cat-related calls to council per 1000 residents and cat impoundments. Leadership of AMOs in this process was identified as a key factor in achieving significant decreases in intake for substantially fewer cats sterilized per capita than in our program (an average of 4.3 versus 27.8 cats/1000 residents per year).

In our pilot, we met with the council animal management team and encouraged their involvement in working collaboratively with the RSPCA Qld and AWLQ outreach teams to notify them of nuisance issues we could assist with to prevent impoundments, e.g., by helping owners/carers with sterilization and containment for their cats. However, AMOs continued to independently bring in trapped cats which prevented an assistive approach being implemented for problematic cats causing complaint calls to council. Initially, the council prohibited in-kind support, but over time, support from AMOs increased and was particularly noticeable in the fourth year after the program successes became evident, complaints to council about the program did not materialize, and there was better understanding of the program.

In the Victorian study, AMOs reported increased job satisfaction associated with an assistive approach, in part because they were now able to better help residents resolve cat-related issues as well as assist the people caring for problematic cats [6]. In a report from Florida, USA, it was noted that when the government shelter shifted from a focus on enforcement to community outreach and an assistance model, AMOs were the most resistant to the new approach [56]. Those who could not adapt were reassigned to different roles. The study emphasized the critical need for comprehensive changes in processes and attitudes across all levels of local government to successfully adopt a community outreach model [56]. The Victorian study also identified that stakeholder engagement at all levels of local government, and particularly AMOs, were critical success factors [57]. In contrast, our program was led by APWF, a not-for-profit, research and advocacy organization, with strong support from RSPCA Qld and subsequent support from AWL Qld.

Engaged AMOs facilitate making a targeted sterilization program more resource effective by targeting the cats most at risk of impoundment, thereby decreasing the number of cats that need to be sterilized to get a measurable decrease in cat intake. Given the cost of sterilizations and the current shortage of veterinary and support staff [58], involvement of AMOs to maximize efficiency is highly recommended. Based on data from Banyule, Victoria and USA, if AMOs had been fully engaged to assist with sterilizing cats most at risk of being impounded or producing unwanted kittens, a similar decrease in intake, euthanasia and cat-related calls could have been achieved with sterilizing 5-10 cats/1000 residents per year instead of 27.8 cats/1000 residents as in our program. This would reduce annual costs and make Community Cat Programs a more feasible investment for cities with populations of 200,000 to over 1 million residents. Development of training programs for AMOs, including case studies that highlight how an assistive approach over time can decrease their workload associated with problematic sites, as well as increase their job satisfaction, would be beneficial and likely accelerate a shift from an enforcement to an assistive approach [6].

### 4.2. Characteristics of Cats Sterilized

In our study, 74% (227) of the 308 cats sterilized were considered owned, which is consistent with recent modelling data from the UK identifying pet cats as the primary contributor to the stray cat population [59]. To stabilize or reduce stray cat numbers, sterilization rates for owned cats of 95% and 98%, respectively, were deemed necessary. This conclusion was based on the assumption that pet cats make up 92% of the urban cat population. In Australia, using 2016 estimates of 3.3 million pet cats and 0.7 million stray cats [60], pet cats represent about 79% of the total cat population, with stray cats (including semi-owned and unowned) accounting for approximately 21%. There are now a substantial number of reports in the literature documenting successful management of free-roaming cats by sterilization programs that target largely owned and semi-owned cats [6,61]. Due to the higher proportion of semi-owned stray cats in Australia, as well as in other countries such as the USA [1,21,54,60], sterilization programs must also focus on these populations. This is particularly important since most of these cats are not sterilized [23,62,63]. In our program, 20% of the cats sterilized were semi-owned, and half remained semi-owned at three multi-cat sites (colonies) and half became owned at the time of sterilization and microchipping. A situational analysis undertaken prior to the start of the Community Cat Program found that 35% of residents in the target suburbs were cat owners and 91% of their cats were sterilized, while 3% of residents were semi-owners. In that study, it was calculated that the two populations contributed approximately equal numbers of kittens annually to the cat population [64]. Availability of free sterilization was a major reason for taking ownership along with an increased attachment over time, a sense of responsibility for the cats and wanting to “do the right thing” [65]. 

#### TNR and RTF-Barriers and Benefits

Trapping, sterilizing, and returning of wandering cats to where they live in cities and towns in Australia is illegal based on biosecurity legislation, containment legislation, and legislation related to abandonment, although the latter has not been tested in court. In our project, we could sterilize cats that remained semi-owned, and return healthy impounded cats that were difficult to adopt back to their home location because of a research permit issued by the Department of Agriculture and Fisheries which allows both TNR and RTF. In our study, only 12% of cats (*n* = 36) remained semi-owned, being cared for by the original carer (*n* = 34), or were returned to their home location after impoundment as strays (*n* = 2). These were sterilized, microchipped, and ear-tipped (RTF cats). There were no sites that we were aware of where cats were solely obtaining food unintentionally from humans, for example, a food bin—classified as unowned domestic cats based on RSPCA Australia recommendations [31]. These cats appear to be rare because generally a compassionate person starts to feed them.

Our permit is the only one the authors are aware of being issued in Australia, and in some other states such as Victoria, there is no provision for such a permit (per com Animal Welfare Victoria). However, TNR is practiced surreptitiously in cities and towns across Australia [22,66,67] and is reported to decrease cat numbers by 30% at the targeted sites within 2 years [22,66]. This is lifesaving for the cats and protects the mental wellbeing of shelter staff, and also of the people caring for them in the community. TNR is vehemently opposed by some environmentalists as evidenced by a grant reviewer of a major Australian government granting body [68] responding to our submission for this project: “I do not wish to see any TNR trial endorsed through the ARC. As our lead research organization, responsible for setting the standards on Australian research quality, this project should not be supported. We have already lost 10% of our native mammal species and a further 20% are threatened, with predation by feral cats and red foxes identified as the principal agent of this loss”. Regarding impacts of cats on wildlife, despite claims based on theoretical calculations of a very large number of native animals killed by pet cats [69,70], population studies have not documented a measurable effect of cats on native birds or mammals in urban or peri-urban areas, or small rural towns such as Rosewood, with habitat loss identified to be the most significant factor [71,72,73].

More consideration also needs to be given to the now well-documented negative mental health impacts on compassionate people who care about animals. For example, carers who feed stray cats, shelter, and council staff tasked with euthanizing healthy and treatable animals as well as AMOs required to continually trap cats, often from multi-cat sites, and transport them for euthanasia [6,33,51]. As stated at a 2020 federal government inquiry into the problem of feral and domestic cats in Australia, AMOs “spend their time trapping cats, then become a taxi for a cat killing program because there is no room left for them [at the shelter]” [74]. In shelters where there is a commitment to low euthanasia rates, timid and fearful stray cats are often held for weeks to months in an attempt to socialize and rehome them. When that fails, the negative impact of their subsequent euthanasia on shelter carers can be massive. In view of the profound mental health impacts including increased risk of suicide, it is recommended that legislative barriers are removed so that cats can be legally sterilized even if their caretakers cannot take ownership and RTF be permitted, particularly in areas where there are no species of conservation concern or scientific proof provided of a negative effect of cats on local wildlife populations.

### 4.3. Cat Admissions

Cat intake decreased by 54% by the end of the first full year of the program and further decreased by 52% and 60% in Years 2 and 3, respectively. In the Banyule program, total city-wide cat admissions decreased from 9.4 to 3.6 cats/1000 residents over 8 years. In comparison, in just 3 years of the Rosewood program, intake decreased from 17.6 cats/1000 residents, which was more than double the state and Australian average (7 cats/1000 residents [24]), to less than the average (6 cats/1000 residents) in the last year.

Although intake in the non-target suburbs decreased by 23%, this was primarily due to a decrease in owner-surrendered adult cats. This first occurred during the COVID-19 pandemic and then continued as intake was actively restricted. The concept of “capacity for care” refers to the practice where the intake to shelters is limited to the number that can be provided with adequate care, ensuring that each animal receives the necessary resources and attention to maintain their well-being [75]. This approach is crucial in preventing overcrowding in shelters, which can lead to stress, illness, and reduced chances of adoption for the animals [76]. Because the Ipswich shelter was obligated to accept stray cats under the council contract, marked restriction of entry of owned cats was the only way to manage intake numbers. 

Kitten intake decreased in Rosewood by an average of 43% over the last 2 years of the program compared to a decrease of 2% in the non-target areas. Prior to implementation of the Community Cat Program, a situational analysis was performed to understand cat caring behaviors and preferences and priorities for management of cats. When residents were asked “How important are the following to you when your council is deciding the best type of management program to manage unowned stray cats in your suburb?”, the highest support was for stopping kittens being born (94% of respondents) [77].

With the change in contracted service provider, there was a change in source of stray cats, with a marked decrease in stray cats being received from the AMOs and a proportional increase in stray cats from members of the public. Council policy was to lend cat traps to residents to trap nuisance or stray cats. To restrict entry of poorly socialized stray cats to facilitate adequate holding time for socialization, trap cage loans were restricted to 5–20 per week, depending on capacity in the shelter at the time. Research shows that adoptability cannot be determined for timid, shy or fearful cats in less than three days, and that 2–4 weeks or longer is required for some cats to adjust to their environment and exhibit behaviors that are more indicative of their true temperament. This time also allows for behavior modification to improve socialization and hence adoptability [78,79]. However, this can lead to long lengths of stay in the shelter which increases risk of disease. When shelter intake exceeds the capacity to rehome cats, there is an increase in euthanasia rates and deaths from infectious diseases due to overcrowding [75,80,81]. This situation highlights the critical need for effective population management strategies to prevent breeding, and other assistive strategies to reduce intake of healthy and treatable cats and kittens into pounds and shelters, to benefit animal and human welfare [79]. It also emphasizes the important role of RTF for trapped cats who do not respond well in a pound or shelter environment, and can be returned to their home location rather than being euthanized, thereby also benefiting human wellbeing.

The biggest decreases in cat admissions and euthanasia are likely to occur when sterilization programs are combined with an increased focus on pet retention programs to assist people to keep their pets [82], for example, through assistance with veterinary costs, impoundment fines, pet food, litter, excess cat permits and cat containment. Cat food was provided for some of the carers of multiple cats after one carer indicated she would need to surrender some of the timid cats because she could not afford to feed them. We committed to providing food and asked her to continue socializing timid cats with the view to rehoming some if they were socialized the following winter when the shelter was at lower capacity. However, to date, she has kept the cats. Pet food costs for low-income carers are substantial, but welfare organizations are often able to provide donated food, as occurs in our program. In contrast, the human and monetary costs are substantial for shelters and pounds of admitting these cats, which are often poorly socialized.

### 4.4. Outcomes for Cats

#### 4.4.1. Return to Owners

Notably, after the Banyule city implemented a free cat sterilization, microchipping, and registration program, the proportion of impounded cats returned to owners increased from 6% to 16% over 8 years [6]. In our study, the proportion of cats reclaimed by owners in the last year represented a small fraction of intake in the target and non-target areas (10%), but was double the average for Australia (5%) [24]. Although higher than reported for USA (2%) [83,84,85], it is markedly lower than the proportion of dogs reported to be reclaimed in Australia (48%) [86]. Over time, the proportion of cats returned to owners would be expected to increase as the proportion of microchipped cats in the community increases. Some local government areas achieve higher reclaim rates for cats (>30%) when they are committed to finding the owner [24,25]. Free community microchipping days at parks and special events for dogs are successful, but because of the challenges of containing cats, including that many cat owners in disadvantaged areas do not have a suitable carrier, cats are not as likely to be microchipped. Other ways of increasing the proportion of owned cats that are microchipped include through Community Cat Programs, where microchipping occurs along with free and affordable sterilization. Other options are microchipping by AMOs when visiting properties.

#### 4.4.2. Adoptions

The proportion of intake that was adopted increased in both the target area and the non-target areas and averaged 81% and 73%, respectively, over the last 2 years of the program. The increase in proportion of intake adopted in Rosewood likely reflects that fewer poorly adoptable cats were being admitted from the target areas. The increase in proportion adopted from the non-target suburbs may have reflected a redeployment of shelter resources because total shelter intake of cats decreased as a result of sequential expansion of the sterilization program into other high-intake suburbs (areas excluded from the data for this study). Decreasing cat intake, especially of shy and timid cats which have longer lengths of stay in shelters and higher risk of euthanasia, frees up shelter resources to help other cats have shorter stays and better outcomes. Actual numbers adopted decreased in the target area, also reflecting decreased intake of cats and fewer cats in need of rehoming. Shelter costs for caring for cats until adoption, including sterilization, microchipping and vaccination, are reported to be A$1000 for an average time to rehoming of 30 days [6,48]. Thus, reducing the number of cats in shelters reduces shelter costs, enabling this funding to be redirected to support people to care for their cats, along with sterilization programs to prevent impoundments from nuisance behaviors and surrender of unwanted kittens.

Although in the study period no cats were adopted as barn or working cats in Rosewood, this is being incorporated in the program at other sites in the city of Ipswich and is being successfully used in the USA for multi-cat sites that need to be relocated [87,88,89]. We have previously documented the value of cats at a dairy farm in Rosewood, where they were reported to prevent thousands of dollars in damage to electrical wiring by rodents and obviated the use of toxic baits [90]. A working or barn cat program is lifesaving for cats that are used to living outside and have little chance of being adopted because they are timid or shy around humans [91]. These cats can be valuable to farmers, and in other situations like horse barns, where there are food stores that attract rodents [92,93].

#### 4.4.3. Euthanasia

The average euthanasia rate in 2018–2019 reported for Australia was 33%, and a quarter of council-operated facilities receiving more than 50 cats a year euthanized 70% to 100% of cats and kittens [24]. In our study, average baseline euthanasia rate in the target area (28%) was similar to the reported Australian average for the same time period. After implementation of the sterilization program, euthanasia decreased from baseline by 85% in the last year from 4.8 cats to 0.6 cats/1000 residents, and euthanized cats constituted an average of 10% of intake. In contrast, in the non-target areas, euthanasia decreased by 48% from 1.6 cats to 0.7 cats/1000 residents, and euthanized cats constituted an average of 13% of intake.

In USA, RTF is also being increasingly used to avoid the negative impact on job satisfaction and mental health of staff having to kill healthy and treatable cats and kittens that are not readily adoptable. After the introduction of RTF programs, cat and kitten euthanasia rates significantly decreased by 67% (from 70% to 23%) [94,95]. Additionally, impound rates decreased by 29%, the incidence of respiratory disease within shelters fell by 99%, and the number of dead cats collected from streets decreased by 20%. The RTF approach is gaining momentum in the USA, with 6.6% of cats returned to the field in 2023, accounting for 12.6% of the stray cat intake [83]. Both cats that were RTF in Rosewood were found by members of the public the following day, taken to the local veterinary clinic and were again impounded. In hindsight, the RTF program would have benefited if information was available to residents in the surrounding area to leave the cats if they appear healthy. Although only two cats were RTF in Rosewood, shelter staff are very supportive of this outcome for cats. When staff are highly committed to minimizing euthanasia, it is mentally very traumatizing if they have worked with cats for 2 or more weeks to socialize them, only for a decision being made that they are not likely to be adoptable and have to be euthanized. Given the well-documented human cost of euthanizing healthy cats [6,9,26,27,28,29,51], including increased risk of suicide, and the lack of documented effects of cats on urban wildlife populations [71,72,73,96], it is strongly recommended that that RTF be legalized as soon as possible. To protect native species of conservation concern in urban and peri-urban areas as well as rural towns, there is an urgent need to map these species across Australian cities and towns, and where they are present, implement targeted strategies to protect them. For example, in addition to protecting habitat, where appropriate, it is recommended that barrier fencing be erected around these sites and/or residents be assisted to install cat proof fencing and to contain their dogs at night.

It is recommended that where cats remain semi-owned but are sterilized and microchipped, or are difficult to adopt, healthy cats who are returned to their home location (RTF), that on the microchip database, secondary contacts such as a rescue group, animal welfare organization, or business are recorded. If concerns arise about the cat’s health, these can then be addressed through the organization, and if issues arise about the cat’s return after sterilization, the organization can assist with alternatives, e.g., placement as a working cat in a less sensitive area. Ideally, the carer’s contact details are also recorded either on the microchip database or with the secondary organization, as occurred in our project.

### 4.5. Cat-Related Complaints and Financial Implications

Cat-related calls decreased by 30% in the last 2 years of the program compared to 16% in the non-target suburbs. The city of Banyule study reported that it cost on average A$290 for each cat-related call that resulted in an AMO trapping a cat, plus an additional fee of A$150 per cat, regardless of source type, which was paid to the contracted shelter to take the trapped cats [6]. The savings to council were over five times the program’s cost. In contrast to Banyule, the city of Ipswich requested that residents hire and pick-up a trap cage from the pound. However, many of the cats being impounded were picked up by AMOs, resulting in a cost to council. Because the city of Ipswich has a 3-year contract with service providers to manage cats after impoundment (with two one-year extensions), a benefit in reducing intake can be realized by the service provider for the duration of the contract. While staff costs may not be reduced if key staff are retained to keep the facility open and operational, reduced intake enables the organization to put more resources into socializing timid and fearful cats in the shelter to prevent their euthanasia, and redirect staff and funding for sterilization and microchipping of cats in the community. Although financial benefit to council during the contract period is mainly related to staff labor and vehicle costs associated with cat-related calls, increased job satisfaction of AMOs and increased resident satisfaction evidenced by fewer cat-related calls are other benefits. The situational analysis performed prior to the study found that only 29% of respondents were satisfied with current local council management of stray and wandering cats [77].

The benefit of reduced cat impoundments is realized by the council when a new contract is negotiated based on anticipated intake. However, when a service provider invests in programs to reduce intake of animals, municipal contracts need to reflect this investment in their contract funding. This is because these programs need to be ongoing, and therefore the contract should not just be based on number of animals impounded in the years prior to the new contract, but also on the cost to maintain Community Cat Programs and other assistive programs.

Assistive cat management is required long-term to maintain low numbers of free-roaming and impounded cats. The median length of tenancy in Queensland is 17.5 months and on average, 6% of homeowners sell their property each year [97,98]. These statistics reflect the mobility trends in the housing market within the region, and consequently, the potential for unsterilized cats and kittens to move into the area with new tenants or homeowners. On average, one fertile female cat produces five kittens per year [99] and there will be a rapid rebound in the population if management is suspended. This potential for rapid population regrowth highlights the importance of effective cat population management strategies being maintained long-term [100], and the cost of these strategies need to be reflected in municipal contracts with service providers. With cat sterilization programs, the number of cats needing sterilization in a given area decreases over time, thereby reducing overall costs [101]. This cost-effectiveness is sustained if consistent cat population management practices are maintained [101,102].

### 4.6. Costs of Community Cat Programs

Cost and accessibility of veterinary services for sterilizations are key factors for implementing Community Cat Programs. Costs for sterilization are substantial, given the number of cats to be sterilized, and vary greatly depending on location and collaborators. However, they are less than the costs of “business as usual” [6]. We were very fortunate and grateful that after the initial seed funding from PetStock, Fondation Brigitte Bardot funded the sterilizations and that RSPCA Qld agreed to the amount the Fondation would pay (A$46.89). RSPCA Qld also had two experienced high-volume, high-quality veterinary surgeons for the sterilizations who were supported by a highly experienced and efficient team of veterinary nurses. This team sterilized up to 68 cats in one day between approximately 8.30 am and 2 pm after expansion of the program to other suburbs. By decreasing the time per surgery, it decreases the unit price per cat. Going forward, there are opportunities and responsibilities for both the large welfare organizations and veterinary schools to train veterinarians in high-volume, high-quality sterilization surgery techniques to reduce sterilization costs for cats in the community.

Human resource costs to employ community liaison officers, as occurred in our program, would largely be eliminated if AMOs were fully engaged in providing community assistance, by finding owners and carers of cats associated with nuisance complaints and transporting cats for sterilization, rather than trapping and transporting cats for impoundment. Some staff time will be required for booking cats for surgery.

Our program and two other effective programs have offered free sterilization and microchipping, which is more costly than providing sterilization at a reduced cost [6,61]. There is a place for reduced cost programs, but they must also be supported by free services to target those most at need, often those caring for multiple cats. When we trailed A$50 and A$49/cat cost for sterilization, the number of cats booked immediately decreased to only 30% of the usual number, but numbers rebounded once the charge was removed. Imposing a financial barrier likely excludes those people with cats most at risk of having unwanted kittens or being impounded, and therefore, risks limiting the success of the program.

### 4.7. Recommendations for Legislative Changes to Support Solutions to Free-Roaming Cats

Current state and local government laws relating to domestic cat management reflect a lack of understanding of the causes and hence effective solutions. For example, there is a belief that free-roaming cats are mainly the result of irresponsible cat owners, and therefore the issue needs to be managed by legislation and its enforcement. However, for enforcement to be effective, there must be an identifiable owner, and the reality is that many free-roaming cats in urban areas live in low socioeconomic areas where the costs of registration, microchipping and cat-proof fencing are often too prohibitive for cat owners and semi-owners to comply with. Mandated sterilization is also not effective, not because of lack of motivation or responsibility by cat owners, but a result of lack of money—household income is the strongest predictor of whether a cat is sterilized [103,104]. To reduce free-roaming domestic cats, legislation and policy need to reflect an understanding of the true causes of the problem and must pursue solutions that are shown scientifically to be effective. Messaging is needed to help the community and its leaders understand the underlying issues and support evidence-based legislation and policy. Messaging that demonizes cats only leads to legislation and bylaws that are barriers to solving the problem, such as mandated containment and cat limits [105].

#### 4.7.1. Cat Containment

The city of Ipswich has a local bylaw requiring cats to be contained on the owner’s property 24/7 [15,106]. A situational analysis performed prior to the implementation of the free sterilization program found 51% of Ipswich cat owners fully contained their cats, and another 18% of owners contained their cats at night [64]. By contrast, a recent study from NSW [107], which does not require mandatory containment, found 65% of residents fully contained their cats and a further 24% contained them at night, suggesting mandating containment is not effective in increasing cat containment. It also leads to unrealistic expectations in the community that they will not see a wandering cat, resulting in increased cat-related complaints. When implemented, mandated containment increases cat-related complaints, cat impoundments, cat euthanasia, and costs to local governments and shelters [108,109,110].

Based on 2021 census statistics for Rosewood, the individual median weekly income (A$636/week) was below the Australian average, a larger proportion of residents lived in rental accommodation and a higher proportion of households had rent repayments greater than 30% of household income [43]. Cat containment systems often cost between A$700 and A$2000 or higher, making it highly unlikely a low-income household can afford one if it is needed. Because of lack of affordability, mandated containment is a barrier to semi-owners taking ownership of a stray cat, and effectively makes cat ownership illegal for many low-income owners, perpetuating the “it’s not my cat” response.

Cat containment should be encouraged and facilitated, but not mandated. Owners can be messaged to provide their cats with a last “bed-time” meal indoors. At little or no cost to the owner, this method trains the cat to come inside at night when the door outside can then be closed. Night-time containment is effective in protecting wildlife of conservation concern susceptible to cat predation because most are nocturnal in urban areas of Australia [111]. Other options include assisting cat owners with the construction or costs of cat-proof fencing or enclosures. Electronic fencing (hidden fencing) may be less expensive for some properties. It is not subject to restrictions for modifying rental properties, and key components can be relocated to another property. It can also be used around doors or windows to stop “door dasher” cats escaping. If used correctly, electronic fencing is not associated with welfare issues [112] and therefore it should be legalized in states where it is not yet permitted.

Management of cat containment issues by local government AMOs should be first based on an assistive approach, and enforcement only used if there is no resolution of the issues. For example, if a cat-related complaint is made, it may be resolved simply by asking neighbours and door-knocking or using flyers to find the owner or carer, and providing free sterilization and microchipping which reduces the desire to wander searching for unsterilized females. If the problem remains, the solution may lie in assisting with fencing or screens for windows, or cat deterrents like motion-activated water-sprays for the affected resident’s property. Individually or combined, in most cases, these assistive approaches resolve most nuisance issues.

#### 4.7.2. Cat Registration

Queensland state legislation only requires microchipping of cats. Mandatory registration (licensing) of cats was repealed in 2013 because repelling it was considered “to deliver the greatest net benefit to stakeholders, as it yields the greatest potential red tape reduction, and cost savings to local governments and cat owners, without compromising reunification outcomes and euthanasia rates” [113,114]. Local governments in Queensland can still implement their own bylaws requiring registration [114,115]. While Ipswich does not have such a requirement, most Australian states or local governments do mandate annual or lifetime registration and microchipping. The associated fees are cost barriers to taking ownership. For example, in NSW, if a stray cat is acquired that is older than 4 months and is not sterilized, there is an annual permit fee payable (A$96) as well as life-time registration (A$69) [116,117]. These fees apply even if the cat was sterilized, microchipped, and registered immediately at, or soon after, acquisition [118,119]. In NSW, return to owner rates are almost half those in Victoria and Queensland (3% versus 7%) [24]. This might reflect that the state microchip register can be used to identify cat owners who have not paid for registration, creating a financial disincentive to microchipping.

Costs to local governments for managing cats in NSW are approximately 7 to 10 times the income to the state government from registration fees [7,120]. Therefore, it makes little fiscal sense to create cost barriers which discourage cat owners from microchipping and thereby reduce return to owner rates. It is recommended instead that mandatory registration be abolished and microchipping made affordable and included with free or affordable sterilization. The effectiveness of microchips for facilitating reuniting cats with their owners can be increased by sending regular SMS messages or email messages reminding owners to update contact details if they have changed [121].

#### 4.7.3. Cat Limits and Excess Cat Permits

Cat limits (typically 2–4 per household) and the costly permits required to exceed these limits should be abolished. There is no evidence a higher number of cats owned by a household correlates to a higher volume of nuisance calls or public health issues. In fact, an irresponsible owner of one cat may generate more complaints than a responsible owner of ten. Any enforcement concerns should focus on the impact of owned cats, not the number of them. Existing laws prohibiting creating a nuisance or a public health hazard are adequate, and cat limits are unnecessary. Moreover, cat limits and costly permits will not necessarily stop residents from feeding and maintaining “excess” cats, but may well deter them from ever taking full ownership.

If the goal is to reduce the overall cat population in the community, this will be achieved more effectively by providing residents with free or affordable sterilization and microchipping of the cats they are caring for. Owners or semi-owners with multiple cats often need support from councils and animal welfare agencies, not only for cat sterilization, but also with transporting the cats for sterilization and provision of carriers. Once this assistance is available, people with multiple cats will feel they can safely ask for help. In this way, rebound in cat numbers and associated nuisance issues for neighbors can be prevented before they develop.

#### 4.7.4. Mandatory Sterilization

A mandate which cannot be met as a practical matter will never achieve the goal of the requirement. From a public policy perspective, there is little point in requiring owners to sterilize their cats if they cannot afford to do so. All that is created is another disincentive to taking ownership. With costs at a private veterinarian for sterilizing and microchipping a female cat ranging on average from A$300 to A$500, few low-income residents and even many middle-income community members cannot afford cat sterilizations, especially if there are multiple cats involved. The cost of sterilization is the number one barrier, and household income is the strongest predictor of a cat being sterilized [103,104].

Mandatory sterilization, especially if it is new legislation, is costly for state governments to implement and for local government animal management teams to enforce. A free-roaming cat suspected of not being sterilized must be trapped first, then checked for identification. Cats without identification will likely be impounded, incurring further costs for local governments. Instead of mandated sterilization and fines for non-compliance, it is highly recommended free and affordable sterilization be provided by local governments. State governments should provide financial incentives to local governments to provide this necessary service, in collaboration with welfare agencies and rescue groups, particularly to residents on low incomes or who are feeding stray cats. More cats sterilized means fewer free-roaming cats and fewer nuisance or predation issues.

#### 4.7.5. Assistive Legislation

Legislation should be designed to achieve desired outcomes, such as fewer free-roaming cats causing a nuisance or less predation by cats on wildlife. When it comes to cat management, laws that unfairly disadvantage low-income households to the point they cannot comply are self-defeating. Without compliance from all community members, cats will continue to reproduce, roam without restriction, and cause whatever ills the legislation seeks to abate. In summary, it is recommended laws mandating containment, registration, sterilization and cat limits be repealed. Instead, issues related to cats should be managed through an assistive approach and enhanced anti-nuisance and animal welfare legislation. There should be a focus on resolving cat complaints through council-led internal processes, including finding the owners or carers of the cats to help with sterilization, microchipping and containment, and mediating until all parties are satisfied, before escalating to more formal actions. Additionally, sterilization of cats should be budgeted for by state and local governments, and areas targeted with a high cat impoundments or cat-related complaints. Semi-owners need to be assisted, including those unable to assume full ownership. RTF should be legalized to protect human wellbeing. These changes are crucial if cat overpopulation is to be effectively managed and to alleviate the severe negative mental health effects experienced by shelter and local government staff that result from inappropriate legislation and enforcement leading to euthanasia of large numbers of healthy cats and kittens. Ultimately fewer free-roaming cats will benefit native wildlife.

### 4.8. Limitations

Despite the positive outcomes of the Community Cat Program in Rosewood, several limitations should be considered when interpreting the results. Firstly, the rural setting and relatively small population of Rosewood (approximately 3000 residents) may limit the generalizability of the findings to larger urban areas or different geographical regions [43]. The specific socioeconomic and environmental characteristics of Rosewood might have influenced the program’s success, and these factors may not be present in other locations. However, there are now results published for similarly successful programs from other states in Australia with similar populations, and for much larger cities [6,61].

Secondly, the data collection and analysis relied heavily on records from the RSPCA Qld and AWLQ, which may have inherent biases or inaccuracies. For example, the categorization of admission sources and outcomes might have been subject to inconsistencies in data entry or interpretation by shelter staff. Additionally, the exclusion of certain admissions, such as those from veterinary clinics without recorded suburbs of origin, may have led to an underestimation of the baseline or an underestimation or overestimation of the true impact of the program on cat intake and outcomes in the target area. Small numbers of cats entering the shelter in the target area led to substantial fluctuations in numbers.

Thirdly, the study period coincided with several external factors, including the COVID-19 pandemic and repeated flooding events, which substantially affected the operations of local animal shelters and the pound. The closures and changes in service providers during this time likely influenced the intake and outcomes of cats in ways that are not entirely attributable to the Community Cat Program itself, although these would be expected to equally affect the target and non-target areas.

## 5. Conclusions

The findings from the Community Cat Program in a small rural town in Australia demonstrate that free-roaming domestic cats can be effectively managed through targeted initiatives that provide free cat sterilization and assist the community to care for their cats. By providing free cat sterilization, microchipping, preventative veterinary care, and other supportive measures, the program significantly reduced cat intake into the shelter by 60% and euthanasia by 85% in the third year. Additionally, the number of cat-related calls to local councils decreased by 39%, indicating improved community satisfaction. These results highlight that non-lethal management strategies can effectively reduce cat intake while promoting animal welfare and reducing the psychological burden on shelter staff. They will also reduce the risk to wildlife. The program’s success suggests that similar approaches could be beneficial in other regions facing challenges with free-roaming cats in urban and peri urban areas, as well as rural towns.

Implementing targeted sterilization programs requires collaboration among animal welfare organizations, local governments, and the community. This program’s success was facilitated by partnerships with RSPCA Qld, AWLQ, and funding from various sources. These collaborations provided preventative care for the cats and assisted the community in improving care for their cats. The study supports a shift from traditional trap-adopt or euthanize methods to more humane and sustainable approaches, aligning with the One Welfare framework that aims to balance and optimize the well-being of animals, humans, and their environments. Future efforts should focus on expanding such programs, securing sustainable funding, and fostering community engagement to create lasting solutions for managing free-roaming cat populations. Although sterilizing and returning cats that remain semi-owned, as well as RTF, were small components of the study, they are very important in protecting human wellbeing and have been embraced by shelter staff. Therefore, state and local government legislation across Australia urgently needs to be amended to allow these approaches to be incorporated in sterilization programs aimed at decreasing free-roaming cats and the issues they cause. These methods will help lessen exposure of staff to the serious negative mental health impacts of euthanizing healthy cats and kittens. Where present, other cost barriers for semi-owners to take ownership of cats such as mandated containment, cat limits, mandatory sterilization, and registration (licensing) should be removed.

## Data Availability

The original contributions presented in the study are included in the article, further inquiries can be directed to the corresponding author.

## Appendix B

**Table A1:** (**a,c**) Sources of cat admissions into RSPCA Queensland and AWL Queensland shelters from 2017–2023 in (**a**) the target suburb Rosewood and (**c**) non-target areas (MOP = member of public). Stray intake sources are given in (**b**,**d**). From August 2020 to 2023 a total of 94 cats/1000 residents were sterilized (308 cats).

	Baseline Years							COVID-19			Community Cat Program (August 2020 to End 2023)								
Sources/Year	2017		2018		2019			2020			2021			2022			2023		
**a. Target Suburb**	**No.**	**% ^1^**	**No.**	**% ^1^**	**No.**	**% ^1^**	**Avg.**	**No.**	**% ^1^**	**%∆ ^2^**	**No.**	**% ^1^**	**%∆ ^2^**	**No.**	**% ^1^**	**%∆ ^2^**	**No.**	**% ^1^**	**%∆ ^2^**
Stray: council + MOP	34	59%	25	71%	50	91%	**36**	29	74%	−21%	20	87%	−45%	24	100%	−35%	20	100%	−45%
Owner surrender	23	40%	5	14%	4	7%	**11**	8	21%	−25%	2	9%	−81%	0	0%	−100%	0	0%	−100%
Ambulance	1	2%	3	9%	1	2%	**2**	2	5%	20%	1	4%	−40%	0	0%	−100%	0	0%	−100%
Shelter offspring	0	0%	2	6%	0	0%	**1**	0	0%	−100%	0	0%	−100%	0	0%	−100%	0	0%	−100%
**Total ^3^**	**59**		**35**		**55**		**50**	**39**		**−21%**	**23**		**−54%**	**24**		**−52%**	**20**		**−60%**
**b. Stray Intake in Target Suburb**																			
*Stray council*	*34*	*100%*	*24*	*96%*	*35*	*70%*	***31***	*8*	*28%*	*−74%*	*0*	*0%*	*−100%*	*3*	*13%*	*−90%*	*3*	*15%*	*−90%*
*Stray MOP*	*0*	*0%*	*1*	*4%*	*15*	*30%*	***5***	*21*	*72%*	*294%*	*20*	*100%*	*275%*	*21*	*88%*	*294%*	*17*	*85%*	*219%*
**Total ^3^**	**34**		**25**		**50**		**36**	**29**		**−20%**	**20**		**−45%**	**24**		**−34%**	**20**		**−45%**
	**Baseline Years**							**COVID-19**			**Control**								
**Sources/Year**	**2017**		**2018**		**2019**			**2020**			**2021**			**2022**			**2023**		
**c. Non-Target Areas**	**No.**	**% ^1^**	**No.**	**% ^1^**	**No.**	**% ^1^**	**Avg.**	**No.**	**% ^1^**	**%∆ ^2^**	**No.**	**% ^1^**	**%∆ ^2^**	**No.**	**% ^1^**	**%∆ ^2^**	**No.**	**% ^1^**	**%∆ ^2^**
Stray: council + MOP	711	82%	741	73%	723	72%	725	611	72%	−16%	756	80%	4%	712	89%	−2%	623	84%	−14%
Owner surrender	90	10%	157	15%	123	12%	123	113	13%	−8%	62	7%	−50%	8	1%	−94%	28	4%	−77%
Ambulance	46	5%	79	8%	71	7%	65	33	4%	−49%	67	7%	3%	46	6%	−30%	44	6%	−33%
Humane officer	6	1%	19	2%	74	7%	33	67	8%	103%	16	2%	−52%	10	1%	−70%	24	3%	−27%
Euthanasia request	18	2%	10	1%	18	2%	15	16	2%	4%	24	3%	57%	17	2%	11%	18	2%	17%
Transfer In	1	0%	4	0%	1	0%	2	0	0%	−100%	0	0%	−100%	0	0%	−100%	7	1%	250%
Shelter offspring	0	0%	5	0%	0	0%	2	6	1%	260%	9	1%	440%	6	1%	260%	0	0%	−100%
**Total ^3^**	**872**		**1015**		**1010**		**966**	**846**		**−12%**	**940**		**−3%**	**803**		**−17%**	**744**		**−23%**
**d. Stray Intake in Non-Target Areas**																			
*Stray council*	*605*	*85%*	*672*	*91%*	*511*	*71%*	***596***	*351*	*57%*	*−41%*	*320*	*42%*	*−46%*	*15*	*2%*	*−97%*	*95*	*15%*	*−84%*
*Stray MOP*	*106*	*15%*	*69*	*9%*	*212*	*29%*	***129***	*260*	*43%*	*102%*	*436*	*58%*	*238%*	*697*	*98%*	*440%*	*528*	*85%*	*309%*
**Total ^3^**	**711**		**741**		**723**		**725**	**611**		**−16%**	**756**		**4%**	**712**		**−2%**	**623**		**−14%**

^1^ Percentage of total admissions; ^2^ Percentage change from baseline average; ^3^ Total number of admissions and percent change from total average baseline admissions.

**Table A2:** (**a**,**c**) Sources of adult cat admissions into RSPCA Queensland and AWL Queensland shelters from 2017–2023 in (**a**) the target suburb Rosewood and (**c**) non-target areas (MOP = member of public). Stray intake sources are given in (**b**,**d**). From August 2020 to 2023 a total of 94 cats/1000 residents were sterilized.

	Baseline Years							COVID-19			Community Cat Program Years (August 2020 to End 2023)								
Sources/Year	2017		2018		2019			2020			2021			2022			2023		
**a. Target Suburb**	**No.**	**% ^1^**	**No.**	**% ^1^**	**No.**	**% ^1^**	**Avg.**	**No.**	**% ^1^**	**%∆ ^2^**	**No.**	**% ^1^**	**%∆ ^2^**	**No.**	**% ^1^**	**%∆ ^2^**	**No.**	**% ^1^**	**%∆ ^2^**
Stray: council + MOP	32	70%	21	75%	29	85%	*27*	13	72%	−52%	6	100%	−78%	19	100%	−30%	9	100%	−67%
Owner surrender	13	28%	3	11%	4	12%	*7*	4	22%	−40%	0	0%	−100%	0	0%	−100%	0	0%	−100%
Ambulance	1	2%	3	11%	1	3%	*2*	1	6%	−40%	0	0%	−100%	0	0%	−100%	0	0%	−100%
Shelter offspring	0	0%	1	4%	0	0%	*0*	0	0%	N/A	0	0%	−100%	0	0%	−100%	0	0%	−100%
**Total ^3^**	**46**		**28**		**34**		**36**	**18**		**−51%**	**6**		**−84%**	**19**		**−49%**	**9**		**−76%**
**b. Stray Intake in Target Suburb**																			
*Stray council*	*31*	*100%*	*20*	*95%*	*22*	*76%*	*24*	*7*	*54%*	*−71%*	*0*	*0%*	*−100%*	*3*	*16%*	*−88%*	*1*	*11%*	*−96%*
*Stray MOP*	*0*	*0%*	*1*	*5%*	*7*	*24%*	*3*	*6*	*46%*	*125%*	*6*	*100%*	*125%*	*16*	*84%*	*500%*	*8*	*89%*	*200%*
**Total ^3^**	***31***		***21***		***29***		***27***	***13***		***−52%***	***6***		***−78%***	***19***		***−30%***	***9***		***−67%***
	**Baseline Years**							**COVID-19**			**Control**								
**Sources/Year**	**2017**		**2018**		**2019**			**2020**			**2021**			**2022**			**2023**		
**c. Non-Target Areas**	**No.**	**% ^1^**	**No.**	**% ^1^**	**No.**	**% ^1^**	**Avg.**	**No.**	**% ^1^**	**%∆ ^2^**	**No.**	**% ^1^**	**%∆ ^2^**	**No.**	**% ^1^**	**%∆ ^2^**	**No.**	**% ^1^**	**%∆ ^2^**
Stray: council + MOP	448	82%	455	72%	427	71%	*443*	384	71%	−13%	428	77%	−3%	351	86%	−21%	348	84%	−22%
Owner surrender	46	8%	97	15%	71	12%	*71*	55	10%	−23%	36	6%	−50%	8	2%	−89%	14	3%	−80%
Ambulance	31	6%	57	9%	47	8%	*45*	29	5%	−36%	54	10%	20%	32	8%	−29%	20	5%	−56%
Humane officer	6	1%	11	2%	41	7%	*19*	49	9%	153%	7	1%	−64%	3	1%	−84%	11	3%	−43%
Euthanasia request	17	3%	9	1%	18	3%	*15*	15	3%	2%	23	4%	57%	10	2%	−32%	17	4%	16%
Transfer In	1	0%	0	0%	1	0%	*1*	6	1%	800%	0	0%	N/A	0	0%	−100%	3	1%	350%
Shelter offspring	0	0%	0	0%	0	0%	*0*	6	1%	N/A	5	1%	N/A	0	0%	N/A	0	0%	N/A
Agency	0	0%	0	0%	0	0%	*0*	0	0%	N/A	6	1%	N/A	4	1%	N/A	0	0%	N/A
**Total ^3^**	**549**		**629**		**605**		***594***	**544**		**−8%**	**559**		**−6%**	**408**		**−31%**	**413**		**−31%**
**d. Stray Intake in Non-Target Areas**																			
*Stray council*	*396*	*88%*	417	*92%*	*297*	*70%*	*370*	*248*	*65%*	*−33%*	*204*	*48%*	*−45%*	*10*	*3%*	*−97%*	*54*	*16%*	*−85%*
*Stray MOP*	*52*	*12%*	38	*8%*	*130*	*30%*	*73*	*136*	*35%*	*85%*	*224*	*52%*	*205%*	*341*	*97%*	*365%*	*294*	*84%*	*301%*
**Total ^3^**	***448***		***455***		***427***		***443***	***384***		***−13%***	***428***		***−3%***	***351***		***−21%***	***348***		***−22%***

^1^ Percentage of total admissions; ^2^ Percentage change from baseline average; ^3^ Total number of admissions and percent change from total average baseline admissions.

**Table A3:** (**a**,**c**) Sources of kitten admissions into RSPCA Queensland and AWL Queensland shelters from 2017–2023 in (**a**) the target suburb Rosewood and (**c**) non-target areas (MOP = member of public). Stray intake sources are given in (**b**,**d**). From August 2020 to 2023 a total of 94 cats/1000 residents were sterilized

	Baseline Years							COVID-19			Community Cat Program Years (August 2020 to End 2023)								
Sources/Year	2017		2018		2019			2020			2021			2022			2023		
**a. Target Suburb**	**No.**	**% ^1^**	**No.**	**% ^1^**	**No.**	**% ^1^**	**Avg.**	**No.**	**% ^1^**	**%∆ ^2^**	**No.**	**% ^1^**	**%∆ ^2^**	**No.**	**% ^1^**	**%∆ ^2^**	**No.**	**% ^1^**	**%∆ ^2^**
Stray: council + MOP	3	23%	4	57%	21	100%	**9**	16	76%	71%	14	82%	50%	5	100%	−46%	11	100%	18%
Owner surrender	10	77%	2	29%	0	0%	**4**	4	19%	0%	2	12%	−50%	0	0%	−100%	0	0%	−100%
Ambulance	0	0%	0	0%	0	0%	**0**	1	5%	N/A	1	6%	N/A	0	0%	N/A	0	0%	N/A
Shelter offspring	0	0%	1	14%	0	0%	**0**	0	0%	N/A	0	0%	N/A	0	0%	N/A	0	0%	N/A
**Total ^3^**	**13**		**7**		**21**		**14**	**21**		**54%**	**17**		**24%**	**5**		**−63%**	**11**		**−20%**
**b. Stray Intake in Target Suburb**																			
*Stray council*	*3*	*100%*	*4*	*100%*	*13*	*62%*	**7**	*1*	*6%*	*−85%*	*0*	*0%*	*−100%*	*0*	*0%*	*−100%*	*2*	*18%*	*−70%*
*Stray MOP*	*0*	*0%*	*0*	*0%*	*8*	*38%*	**3**	*15*	*94%*	*463%*	*14*	*100%*	*425%*	*5*	*100%*	*88%*	*9*	*82%*	*238%*
**Total ^3^**	**3**		**4**		**21**		**9**	**16**		**71%**	**14**		**50%**	**5**		**−46%**	**11**		**18%**
	**Baseline Years**							**COVID-19**			**Control**								
**Sources/Year**	**2017**		**2018**		**2019**			**2020**			**2021**			**2022**			**2023**		
**c. Non-Target Areas**	**No.**	**% ^1^**	**No.**	**% ^1^**	**No.**	**% ^1^**	**Avg.**	**No.**	**% ^1^**	**%∆ ^2^**	**No.**	**% ^1^**	**%∆ ^2^**	**No.**	**% ^1^**	**%∆ ^2^**	**No.**	**% ^1^**	**%∆ ^2^**
Stray: council + MOP	263	81%	286	74%	296	73%	*282*	227	72%	−19%	328	86%	16%	361	91%	28%	275	83%	−2%
Owner surrender	44	14%	60	16%	52	13%	*52*	58	18%	12%	26	7%	−50%	0	0%	−100%	14	4%	−73%
Ambulance	15	5%	22	6%	24	6%	*20*	4	1%	−80%	13	3%	−36%	14	4%	−31%	24	7%	18%
Humane officer	0	0%	8	2%	33	8%	*14*	18	6%	32%	9	2%	−34%	7	2%	−49%	13	4%	−5%
Euthanasia request	1	0%	1	0%	0	0%	*1*	1	0%	50%	1	0%	50%	7	2%	950%	1	0%	50%
Transfer In	0	0%	4	1%	0	0%	*1*	6	2%	350%	0	0%	−100%	0	0%	−100%	4	1%	200%
Shelter offspring	0	0%	5	1%	0	0%	*2*	0	0%	−100%	4	1%	140%	6	2%	260%	0	0%	−100%
**Total ^3^**	**323**		**386**		**405**		***371***	**314**		**−15%**	**381**		**3%**	**395**		**6%**	**331**		**−11%**
**d. Stray Intake in Non-Target Areas**																			
*Stray council*	*209*	*79%*	*255*	*89%*	*214*	*72%*	*226*	*103*	*45%*	*−54%*	*116*	*35%*	*−49%*	*5*	*1%*	*−98%*	*41*	*15%*	*−82%*
*Stray MOP*	*54*	*21%*	*31*	*11%*	*82*	*28%*	*56*	*124*	*55%*	*123%*	*212*	*65%*	*281%*	*356*	*99%*	*540%*	*234*	*85%*	*320%*
**Total ^3^**	***263***		***286***		***296***		***282***	***227***		***−19%***	***328***		***16%***	***361***		***28%***	***275***		***−2%***

^1^ Percentage of total admissions; ^2^ Percentage change from baseline average; ^3^ Total number of admissions and percent change from total average baseline admissions.

**Table A4:** (**a**,**b**) Distribution of outcomes of all admissions into RSPCA Queensland and AWL Queensland shelters from 2017–2023 in (**a**) the target suburb Rosewood and (**b**) non-target areas. From August 2020 to 2023 a total of 94 cats/1000 residents were sterilized (308 cats).

a. Target Suburb	Baseline Years							COVID-19			Community Cat Program Years (August 2020 to End 2023)								
Outcome/Year	2017		2018		2019			2020			2021			2022			2023		
	**No.**	**% ^1^**	**No.**	**% ^1^**	**No.**	**% ^1^**	**Avg.**	**No.**	**% ^1^**	**%∆ ^2^**	**No.**	**% ^1^**	**%∆ ^2^**	**No.**	**% ^1^**	**%∆ ^2^**	**No.**	**% ^1^**	**%∆ ^2^**
Reclaimed	2	3%	8	23%	2	4%	4	5	13%	25%	1	4%	−75%	1	4%	−75%	2	10%	−50%
Adopted	40	68%	14	40%	33	60%	29	33	85%	14%	21	91%	−28%	21	88%	−28%	15	75%	−48%
Transfer Out	0	0%	0	0%	5	9%	2	0	0%	−100%	0	0%	−100%	1	4%	−40%	0	0%	−100%
Euthanized	15	25%	11	31%	15	27%	14	0	0%	−100%	1	4%	−93%	1	4%	−93%	2	10%	−85%
In Care	0	0%	0	0%	0	0%	0	1	3%	N/A	0	0%	N/A	0	0%	N/A	1	5%	N/A
Other—Deceased	0	0%	1	3%	0	0%	0	0	0%	N/A	0	0%	N/A	0	0%	N/A	0	0%	N/A
Lost/Escaped/Released by agency	2	3%	1	3%	0	0%	1	0	0%	−100%	0	0%	−100%	0	0%	−100%	0	0%	−100%
**Total ^3^**	**59**		**35**		**55**		**50**	**39**		**−22%**	**23**		**−54%**	**24**		**−52%**	**20**		**−60%**
**b. Non-Target Areas**	**Baseline Years**							**COVID-19**			**Control**								
**Outcome/Year**	**2017**		**2018**		**2019**			**2020**			**2021**			**2022**			**2023**		
	**No.**	**% ^1^**	**No.**	**% ^1^**	**No.**	**% ^1^**	**Avg.**	**No.**	**% ^1^**	**%∆ ^2^**	**No.**	**% ^1^**	**%∆ ^2^**	**No.**	**% ^1^**	**%∆ ^2^**	**No.**	**% ^1^**	**%∆ ^2^**
Reclaimed	60	7%	95	9%	83	8%	79	75	9%	−5%	89	9%	12%	69	9%	−13%	73	10%	−8%
Adopted	504	58%	620	61%	654	65%	593	575	68%	−3%	638	68%	8%	602	75%	2%	528	71%	−11%
Transfer Out	17	2%	14	1%	12	1%	14	2	0%	−86%	18	2%	26%	0	0%	−100%	6	1%	−58%
Euthanized	195	22%	180	18%	171	17%	182	145	17%	−20%	170	18%	−7%	101	13%	−45%	94	13%	−48%
In Care	3	0%	3	0%	6	1%	4	5	1%	25%	4	0%	0%	2	0%	−50%	38	5%	850%
Other—Deceased	18	2%	19	2%	19	2%	19	12	1%	−36%	12	1%	−36%	23	3%	23%	9	1%	−52%
Lost	26	3%	36	4%	22	2%	28	10	1%	−64%	5	1%	−82%	0	0%	−100%	0	0%	−100%
Escaped/Released by agency	1	0%	0	0%	1	0%	1	0	0%	−100%	1	0%	50%	1	0%	200%	1	0%	50%
Returned to protective agency	0	0%	0	0%	3	0%	1	0	0%	−100%	0	0%	−100%	0	0%	−100%	6	1%	500%
Returned home	45	5%	41	4%	33	3%	40	17	2%	−57%	7	1%	−82%	2	0%	−95%	1	0%	−97%
**Total ^3^**	**870**		**1008**		**1004**		**961**	**841**		**−12%**	**944**		**−2%**	**800**		**−17%**	**756**		**−21%**

^1^ Percentage of total admissions; ^2^ Percentage change from baseline average; ^3^ Total number of admissions and percent change from total average baseline admissions.

**Table A5:** (**a**,**b**) Breakdown of reason for cat-related calls to the city of Ipswich from 2017–2023 in (**a**) the target suburb Rosewood and (**b**) non-target areas showing baseline average (Avg.) over three years (2017–2019). From August 2020 to 2023 a total of 94 cats/1000 residents were sterilized (308 cats).

a. Target Suburb	Baseline Years							COVID-19			Community Cat Program Years (August 2020 to End 2023)								
Reason/Year	2017		2018		2019			2020			2021			2022			2023		
	**No.**	**% ^1^**	**No.**	**% ^1^**	**No.**	**% ^1^**	**Avg.**	**No.**	**% ^1^**	**%∆ ^2^**	**No.**	**% ^1^**	**%∆ ^2^**	**No.**	**% ^1^**	**%∆ ^2^**	**No.**	**% ^1^**	**%∆ ^2^**
Loan of Cat Trap	20	91%	12	86%	9	69%	14	12	100%	−12%	5	63%	−63%	10	77%	−27%	7	70%	−49%
Roaming Cat	1	5%	2	14%	1	8%	1	0	0%	−100%	2	25%	50%	2	15%	50%	3	30%	125%
Cat Excess Numbers	1	5%	0	0%	3	23%	1	0	0%	−100%	1	13%	−25%	1	8%	−25%	0	0%	−100%
Cat Enclosure/Fencing	0	0%	0	0%	0	0%	0	0	0%	N/A	0	0%	N/A	0	0%	N/A	0	0%	N/A
Cat Noise Nuisance	0	0%	0	0%	0	0%	0	0	0%	N/A	0	0%	N/A	0	0%	N/A	0	0%	N/A
**Total ^3^**	**22**		**14**		**13**		**16**	**12**		**−27%**	**8**		**−51%**	**13**		**−20%**	**10**		**−39%**
**b. Non-Target Areas**	**Baseline Years**							**COVID-19**			**Control**								
**Reason/Year**	**2017**		**2018**		**2019**			**2020**			**2021**			**2022**			**2023**		
	**No.**	**% ^1^**	**No.**	**% ^1^**	**No.**	**% ^1^**	**Avg.**	**No.**	**% ^1^**	**%∆ ^2^**	**No.**	**% ^1^**	**%∆ ^2^**	**No.**	**% ^1^**	**%∆ ^2^**	**No.**	**% ^1^**	**%∆ ^2^**
Loan of Cat Trap	262	69%	302	81%	248	77%	271	231	80%	−15%	242	78%	−11%	264	77%	−2%	209	79%	−23%
Roaming Cat	97	25%	42	11%	53	16%	64	39	14%	−39%	43	14%	−33%	51	15%	−20%	42	16%	−34%
Cat Excess Numbers	16	4%	26	7%	19	6%	20	16	6%	−21%	10	3%	−51%	14	4%	−31%	10	4%	−51%
Cat Enclosure/Fencing	7	2%	1	0%	2	1%	3	2	1%	−40%	14	5%	320%	12	4%	260%	3	1%	−10%
Cat Noise Nuisance	0	0%	2	1%	0	0%	1	0	0%	−100%	1	0%	50%	0	0%	−100%	0	0%	−100%
**Total ^3^**	**382**		**373**		**322**		**359**	**288**		**−20%**	**310**		**−14%**	**341**		**−5%**	**264**		**−26%**

^1^ Percentage of total admissions; ^2^ Percentage change from baseline average; ^3^ Total number of admissions and percent change from total average baseline admissions.
